# *DICER1* and *DGCR8* in thyroid tumorigenesis: miRNA biogenesis and histopathologic diversity

**DOI:** 10.1530/ETJ-25-0188

**Published:** 2025-11-04

**Authors:** Lia Rodrigues, Rui Sousa Martins, Valdemar Máximo, Paula Soares, Joao Vinagre, Vania Nosé, Sule Canberk

**Affiliations:** ^1^ Institute for Research and Innovation in Health of the University of Porto (I3S), Porto, Portugal; ^2^ Institute of Molecular Pathology and Immunology of the University of Porto (Ipatimup), Porto, Portugal; ^3^ RISE-Health, Department of Pathology, Faculty of Medicine of the University of Porto (FMUP), Porto, Portugal; ^4^ School of Health Polytechnic of Porto (E2S), Porto, Portugal; ^5^ Department of Pathology, Massachusetts General Hospital and Harvard Medical School, Boston, Massachusetts, USA

**Keywords:** thyroid, oncocytic neoplasms, *DICER1*, *DGCR8*, miRNA biogenesis

## Abstract

This review examines the emerging roles of *DICER1* and *DGCR8*, key components of the miRNA biogenesis pathway, in thyroid pathogenesis, with a particular focus on their association with oncocytic morphology. Recent findings have expanded our understanding of DICER1 syndrome and *DGCR8*-related thyroid disorders, revealing a broader spectrum of thyroid lesions associated with mutations in these genes than previously recognised. We analyse the current literature on *DICER1* and *DGCR8* mutations in thyroid pathology, synthesising data from both basic science and pathological studies. The review explores recent findings on oncocytic features in some *DICER1*-mutated thyroid lesions, acknowledging that this association remains under investigation. The manuscript details the molecular mechanisms underlying *DICER1* and *DGCR8* mutations, including their impact on miRNA processing and subsequent effects on gene expression and cellular function. We discuss the diverse range of thyroid lesions associated with these mutations, from benign follicular nodular disease to aggressive carcinomas. The clinical implications of these findings are significant, as recognising *DICER1* and *DGCR8*-related thyroid lesions can lead to improved patient management, including genetic counselling and surveillance for other associated malignancies. We propose an algorithm for identifying *DICER1*-related thyroid lesions, with a focus on oncocytic tumours, to aid clinicians and pathologists in recognising these entities. This emerging field promises to refine the diagnosis, management, and treatment of thyroid disorders associated with miRNA biogenesis pathway alterations, potentially leading to novel diagnostic and therapeutic approaches.

## Introduction

The identification and characterisation of inherited tumour syndromes have expanded significantly in recent decades, with many syndromes still under investigation for formal recognition. Reflecting the growing importance and complexity of these syndromes, the latest World Health Organization (WHO) classification of endocrine tumours introduced a dedicated chapter on familial tumour syndromes (1). This section addresses key syndromes, including PTEN hamartoma tumour syndrome, familial adenomatous polyposis, Carney complex, Werner syndrome, and DICER1 syndrome, as well as the umbrella term ‘not specified: Syndromic Familial Non-Medullary Thyroid Carcinoma (SFNMTC)’, reserving space for yet-to-be formally recognized syndromes. While 90% of thyroid cancers (TC) occur sporadically, 3–9% are familial non-medullary thyroid cancers (FNMTCs), with less than 5% associated with syndromic forms involving well-defined germline driver alterations (2). The remaining 95% of FNMTCs represent a less defined genetic susceptibility group, termed ‘non-syndromic’ (2).

The *DICER1* gene, a key component of the microRNA (miRNA) biogenesis pathway, has been implicated in various benign and malignant lesions across multiple organs (3, 4, 5). Germline mutations in *DICER1* increase susceptibility to conditions including thyroid neoplasms, as part of DICER1 syndrome, also known as autosomal dominant hereditary pleiotropic tumour syndrome. Benign thyroid lesions, such as follicular nodular disease (FND) and follicular thyroid adenomas (FTA), are the most common thyroid manifestations in DICER1 syndrome (6). Thyroid disorders associated with *DICER1* have drawn increasing attention since 2009, when the first report of a *DICER1* mutation in pleuropulmonary blastoma (PPB) was published (7). Subsequently, Rio Frio *et al.* (8), in 2011, highlighted thyroid lesions as common manifestations of DICER1 syndrome by documenting five families with FND and heterozygous *DICER1* mutations. It has since been recommended that early thyroid disease occurrence, particularly in childhood or in association with other organ-specific manifestations, warrants genetic screening and family history evaluation (9, 10).

Although DICER has been more extensively studied, emerging research suggests that another key player in miRNA biogenesis, DGCR8, may also contribute to thyroid tumorigenesis. Barbara Rivera *et al.* (11) identified a pathogenic variant of DiGeorge Syndrome Critical Region Gene 8 *(DGCR8*) in familial FND with schwannomatosis, while Paulsson *et al.* (12) and Rodrigues *et al.* (13) demonstrated *DGCR8*’s involvement in follicular-patterned thyroid carcinomas. Paulsson *et al.* (12) reported *DGCR8* downregulation in follicular thyroid carcinoma (FTC) compared to FTA, and Rodrigues *et al.* (13) suggested *DGCR8* mRNA overexpression in FTA might help maintain normal thyroid morphology, while reduced expression was implicated in dedifferentiation processes of follicular-patterned carcinomas. *DGCR8* mutations were also linked to poorly differentiated thyroid carcinoma (PDTC), indicating a possible role in thyroid gland morphology and tumorigenesis (13).

Our group was the first to report a higher frequency of *DICER1* germline variants in papillary thyroid carcinoma (PTC) cases with oncocytic morphology compared to non-oncocytic counterparts (9). In addition, oncocytic morphology was associated with *DGCR8* mutations in a study by Paulsson *et al.* (12). These findings suggest a close relationship between oncocytic morphology, miRNA biogenesis, and epigenetic processes (14). This review systematically examines the role of *DICER1* and *DGCR8*, two of the main miRNA processing genes regulating miRNA maturation, as central elements in the miRNA biogenesis pathway, emphasising their specific association with oncocytic morphology.

### miRNA biogenesis pathway and its partners

MiRNAs are small, highly conserved non-coding RNAs that regulate gene expression by targeting mRNAs for degradation or translational repression (15). These regulatory processes are critical for cell fate determination and development (15), and their dysregulation has been strongly linked to cancer initiation, progression, and metastasis (5, 16). Depending on their mRNA targets, miRNAs can act as oncogenes (oncomiRs) or tumour suppressors (oncosuppressor miRs) (17, 18). Cancer cells often exhibit suppressed or aberrant miRNA expression, driven by disrupted regulatory feedback loops involving transcription factors and epigenetic mechanisms such as DNA methylation (5, 15, 19, 20).

**Figure 1:**
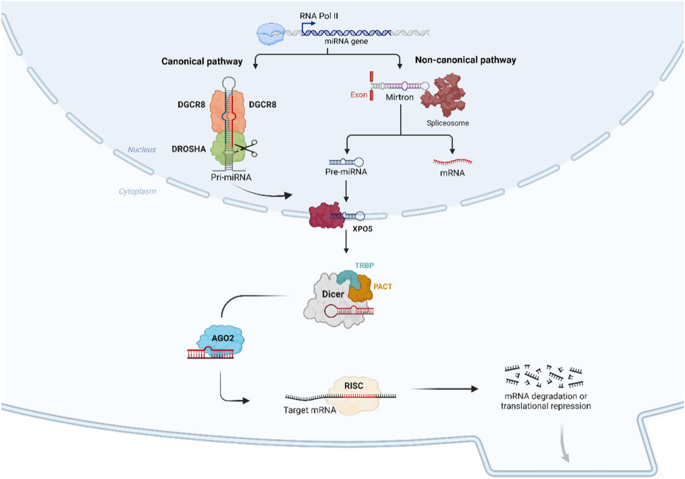
miRNAs can be produced via the canonical pathway, involving the DROSHA–DGCR8 microprocessor complex, or through the non-canonical pathway from mirtrons in the nucleus. After exportation of the pre-miRNA by XPO5, cytoplasmic components such as DICER, TRBP, PACT, and AGO2 will process the pre-miRNA into the mature and active miRNA. Created with BioRender.com.

The miRNA biogenesis pathway includes both nuclear and cytoplasmic processes, as outlined in Fig. 1. In the canonical pathway, DGCR8 is a pivotal nuclear component of the DROSHA-DGCR8 microprocessor complex, which processes primary miRNAs (pri-miRNA) into precursor miRNAs (pre-miRNA) (5, 15). The pre-miRNA is then exported to the cytoplasm by exportin 5 (XPO5), where DICER cleaves it into a ∼20 bp duplex with the help of trans-activation-responsive RNA-binding protein (TRBP). One strand of this duplex is loaded onto an Argonaute (AGO) protein within the RNA-induced silencing complex (RISC), guiding the mature miRNA to its mRNA target for translational repression or degradation (5, 15, 16).

In the non-canonical pathway, pre-miRNAs can arise from short introns (mirtrons) via splicing and debranching, bypassing the need for DROSHA-DGCR8 processing (5, 15). Both DROSHA and DICER require associated RNA-binding proteins (RBPs) for stability and function: DGCR8 stabilises DROSHA, while TRBP supports DICER activity (15). Deficiencies in these RBPs, or their phosphorylation by the MAPK/ERK pathway, can lead to irregularities in miRNA processing and contribute to cancer development (21, 22).

Recent studies have identified a non-canonical nuclear role for DICER, suggesting it may influence oncocytic characteristics in thyroid lesions, highlighting its potential involvement in thyroid tumorigenesis (23, 24, 25). Germline and somatic *DICER1* mutations have been implicated in various thyroid tumours, further supporting the link between miRNA pathway components and thyroid disease (26). Similarly, germline and somatic mutations in *DGCR8* have been associated with thyroid lesions, as demonstrated by Rivera *et al.* (11), Paulsson *et al.* (12), and Rodrigues *et al.* (13). These findings reinforce the critical connection between miRNA biogenesis and thyroid health, where genes with similar function in miRNA maturation, *DGCR8* and *DICER1*, are associated with thyroid lesions.

### From *DICER1* dispersed germline mutations to *DICER1* hotspot somatic mutations

The *DICER1* gene, located on chromosome 14q32.13, encodes a 1922-amino acid protein with 27 exons (27). Mutations in *DICER1* occur in both germline and somatic forms, with germline mutations being dispersed across the gene and predominantly resulting in loss of function (LOF), leading to DICER1 syndrome (3). These germline mutations often involve nonsense, frameshift, or splice site mutations, as well as deletions or intragenic rearrangements, resulting in truncated proteins and reduced RNase III functionality, subsequently lowering miRNA levels (28, 29, 30). Interestingly, germline mutations can also occur in asymptomatic carriers, highlighting variability in clinical presentation.

**Figure 2:**
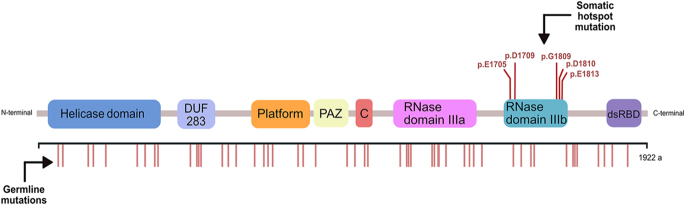
Schematic representation of the *DICER1* protein, showing germline and hotspot somatic mutation locations. DICER is composed of the following domains, from N- to C-terminus of the protein: helicase domain (hel1, Hel2i, and Hel2), DUF283, platform, Piwi-Argonaute-Zwille (PAZ), connector helix I, RNase IIIa, RNase IIIb, and double-stranded RNA-binding domain (dsRBD). The RNase IIIb domain contains the hotspot somatic mutations associated with thyroid lesions, while germline mutations are dispersed throughout the gene.

In contrast, somatic mutations in *DICER1* are predominantly clustered within the RNase IIIb domain (Fig. 2) (2, 31). Mosaicism in this region, characterised by RNase IIIb hotspot mutations, often leads to an earlier onset and multisite disease. This unique pattern requires additional alterations to cause LOF, and these missense hotspot mutations are associated with more severe phenotypes (3, 27, 32). The metal-binding sites of the RNase IIIb domain, particularly at amino acids E1705, D1709, G1809, D1810, and E1813, are frequently affected in syndromic cases (26, 27, 33).

DICER1 syndrome, first characterised in 2009 and recognised in the 2022 WHO classification, is defined as an ‘autosomal dominant tumour predisposition syndrome caused by heterozygous germline pathogenic variants in *DICER1*’ (1). A stepwise model proposed by Khan *et al.* (34) suggests that biallelic *DICER1* mutations increase benign thyroid nodule prevalence, which, over time, may acquire additional genetic alterations leading to malignant transformation. Mouse models demonstrated that homozygous deletion of *DICER1* results in embryonic lethality, underscoring the importance of haploinsufficiency (35). Individuals with germline *DICER1* mutations have a 16-fold increased risk of thyroid tumours (3, 27, 34, 36, 37).

Germline *DICER1* mutations causing LOF occur in approximately 1 in 5,310 to 1 in 12,412 individuals (38). Additional acquired somatic mutations, particularly hotspot missense mutations in the RNase IIIb domain, contribute to thyroid tumorigenesis. Furthermore, other oncogenic events outside the *DICER1* gene, such as mutations in *BRAF*, *NRAS*, and *EIF1AX*, can elevate the risk of thyroid lesions (3, 4).

### How *DICER1* molecular alterations manifest in thyroid pathology

Thyroid lesions are increasingly recognised as critical clinical markers for the early diagnosis of DICER1 syndrome, emphasising the need for proactive surveillance for malignancies in other organs to improve patient outcomes. The presence of distinct histomorphological features in *DICER1*-associated thyroid lesions, especially in early life and even without a family history, should raise suspicion for *DICER1* involvement (26, 39). Pathologists play a key role in identifying these histomorphologies and guiding appropriate molecular testing, which benefits both patient care and familial management.

Initially, *DICER1*-related thyroid manifestations were primarily linked to FND or FTA in syndromic cases (40, 41, 42). However, the recent literature has revealed a phenotypically diverse spectrum of thyroid entities ranging from benign to highly aggressive tumours (8, 39, 40, 43). Unique histomorphological patterns, such as intrafollicular centripetal papillary growth in FND, extensive papillary folds with embedded subfollicles in FTA, and a macrofollicular pattern in both benign and malignant tumours, have been described by Nosé *et al.* (4). Besides these benign lesions, thyroid entities such as non-invasive follicular thyroid neoplasm with papillary-like nuclear features (NIFTP), invasive encapsulated follicular variants of papillary thyroid cancer (IEFVPTC), PTC, and FTC are also associated with germline and somatic *DICER1* mutations (3, 4). Regarding paediatric tumours, *DICER1* mutations have been highly frequent in paediatric low-risk follicular-patterned tumours, IEFVPTC, and FTC (39, 40, 41, 42).

Two aggressive tumour entities have also been linked to *DICER1* mutations and are now recognised in the 2022 WHO classification (1). The first is paediatric PDTC, highlighted by Chernock *et al.* (43), who identified somatic *DICER1* mutations in four out of six cases, predominantly in the RNase IIIb domain; these mutations are less frequently germline, occurring in only one case (43). Nosé *et al.* (4) noted that these paediatric PDTCs lack convoluted nuclei and adult-type molecular alterations, with outcomes varying widely. Supporting this, Yegen *et al.* (44) described a paediatric PDTC with a *DICER1* mutation but no vascular invasion, suggesting a potentially favourable prognosis. In contrast, Ver Berne *et al.* (45) reported a PDTC case with both germline and somatic *DICER1* mutations, characterised by aggressive features such as solid/trabecular growth, central necrosis, and vascular invasion. These findings emphasise the need for further research into *DICER1*’s role in paediatric PDTC.

The second entity, thyroblastoma, was described by Agaimy *et al.* (46), who reviewed eight cases of sporadic malignant teratoid thyroid tumours. These tumours, characterised by somatic *DICER1* hotspot mutations, differ from teratomas or carcinosarcomas and often occur in older individuals without a familial cancer history. Recognised in the 2022 WHO classification, thyroblastoma is now classified as a distinct entity, with germline *DICER1* testing recommended (2). Furthermore, Rooper *et al.* (47) identified *DICER1* hotspot mutations in four malignant thyroid teratomas, now recognised as thyroblastoma (48).

A more recent bi-institutional study identified atrophic changes associated with *DICER1* mutations in follicular-patterned thyroid tumours (26). These changes are characterised by pale, ghost-cell-like features with clear demarcation from adjacent tissue, thickened stroma, and reduced cell viability, distinct from post-fine needle aspiration (FNA) biopsy artefacts (26).

*DICER1* mutations, once thought to be predominantly associated with benign lesions, are now increasingly linked to aggressive cancers in both children and young adults (39). However, documentation of oncocytic morphology remains sparse. Nevertheless, *DICER1* mRNA expression was previously reported as severely downregulated in OCA (then referred to as Hürthle cell carcinoma, HCC), followed by FTC and FTA, when compared to normal thyroid tissue (49).

In Table 1, a comparison between germline and somatic *DICER1* mutations in thyroid disease is outlined, highlighting key differences in morphology, clinical implications, and testing recommendations (3, 4, 9, 50, 51, 52, 53, 54, 55, 56).

**Table 1:** Comparison of germline vs somatic *DICER1* mutations in thyroid disease: morphology, clinical implications, and testing recommendations.

Feature	Germline *DICER1* (DICER1 syndrome)	Somatic *DICER1* (non‐Syndromic)
Inheritance/genetic basis	Autosomal dominant Usually a LOF mutation in one allele + hotspot RNase IIIb mutation in tumour Rarely mosaic	Acquired RNase IIIb ‘hotspot’ mutations only No germline involvement (isolated to the tumour)
Prevalence in thyroid disease	Rare overall, but is the most common *DICER1* driver in paediatric thyroid disease Thyroid disease is the most frequent manifestation of DICER1 syndrome	Small subset of thyroid cancers in adults In paediatrics, can be seen in thyroblastoma or PDTC, more often than in adult PDTC
Age group and clinical history	Typically paediatric/adolescent onset (though adult cases occur) Often with personal/family history of *DICER1*‐related tumours (e.g., PPB, cystic nephroma, SLCT)	All ages (though certain subtypes may appear in paediatrics) Usually no known *DICER1*‐associated tumours in the patient/family
Morphology and pathology	Often multifocal/bilateral nodules, sometimes as FND Typically encapsulated/well‐circumscribed follicular‐patterned lesions (FTA, FVPTC, FTC) Paediatric malignancies can include FTC, FVPTC, and rarely PDTC; benign lesions such as FND and FTA occur in the benign setting	Often unilateral, unifocal Identified in paediatric FTC, but more commonly identified in rare paediatric lesions (thyroblastoma, PDTC), though not all paediatric PDTC is *DICER1* mutated
Co-occurring somatic alterations	Occasional co‐occurrence with *BRAF*, *RAS*, or other thyroid drivers ’Two‐hit’ hypothesis (germline + somatic) is the norm	Typically no common somatic driver alterations (*BRAF*, *RAS*, *TP53* mutations) or *RET/PTC* rearrangements Often the tumour harbours only the *DICER1* ‘hotspot’ mutation
Clinical relevance	High: indicates a hereditary predisposition Genetic counselling + family screening recommended	Limited in routine clinical practice No hereditary risk solely from a somatic *DICER1* finding
Indications for genetic testing	Paediatric thyroid nodule (particularly bilateral, multifocal) or with unusual histology Any age + personal/family history of *DICER1*‐related tumours Confirmed *DICER1* ‘hotspot’ + clinical suspicion	Consider germline testing if a somatic RNase IIIb hotspot mutation is identified in a thyroid lesion, as per DICER1 surveillance guidelines. Del/Dup and RNA analysis may be warranted even in the absence of other syndromic features
Management and surveillance	Initial thyroid ultrasound at 8 years of age, or at diagnosis if it is done between 40 and 50 years of age If a nodule is detected: annual thyroid US If initial scan is normal: repeat every 3 years Consider annual thyroid US for 5 years following chemotherapy or radiotherapy No role for prophylactic thyroidectomy unless clinically indicated Broaden screening to include entire *DICER1*-related tumour spectrum Standard thyroid tumour management based on histotype and staging Family screening guided by personal/family history or syndromic indicators	Standard thyroid tumour management based on histotype and staging No specific *DICER1* family screening unless other syndromic indicators are present

LOF, loss of heterozygosity; PDTC, poorly differentiated thyroid carcinoma; PPB, pleuropulmonary blastoma; SLCT, Sertoli–Leydig cell tumour; FND, follicular nodular disease; FTA, follicular adenoma; FVPTC, follicular-variant papillary thyroid carcinoma; FTC, follicular thyroid carcinoma; US, ultrasound.

For *DGCR8*, the focus has been even more limited, though its molecular role in thyroid tumorigenesis is gaining attention. *DGCR8* mutations disrupt *let*-7 miRNAs, which regulate *RAS* expression, leading to tumorigenesis and poor prognosis (57). *DGCR8* expression levels vary by histotype: FTA shows overexpression, whereas NIFTP and classical PTC exhibit reduced expression (12, 13, 58, 59). The *DGCR8*-mutated cases and subsets of cases with low *DGCR8* expression translate into a specific miRNA profile, correlating with dedifferentiation and tumour progression (12).

The association between *DGCR8* and oncocytic morphology remains underexplored despite emerging evidence from TC cohorts (12). This gap is particularly striking given the relationship between epigenetic changes in oncocytic tumours and miRNA biogenesis mechanisms. These findings show the need for further research to clarify the involvement of *DICER1* and *DGCR8* in oncocytic thyroid lesions. The following chapter will focus on this connection, drawing insights from a comprehensive review of the literature, spanning both basic science and pathology, to highlight critical areas for future exploration.

### Oncocytic morphology observed in *DICER1* and *DGCR8*-altered thyroid tumours: an exploratory perspective on miRNA biogenesis pathways

Oncocytic morphology is defined histologically by cells with abundant, finely and densely granular, eosinophilic cytoplasm due to dysfunctional mitochondrial accumulation (historically ‘Hürthle cell’ change). It occurs across benign and malignant follicular-derived thyroid tumours, including oncocytic adenoma, OCA, and PTC variants such as oncocytic, tall cell, Warthin-like, and hobnail. Oncocytic change alone is not pathognomonic for any tumour type or molecular alteration. In this section, we report its observed frequency in *DICER1-* and *DGCR8*-altered tumours strictly as an observational correlate. At present, no morphologic or molecular criteria reliably distinguish oncocytic change seen in *DICER1*-associated tumours from that in other entities, and we do not propose a diagnostic category of ‘*DICER1*-associated oncocytic morphology’. Any putative relationship remains hypothesis-generating in the absence of mechanistic data.

**Figure 3:**
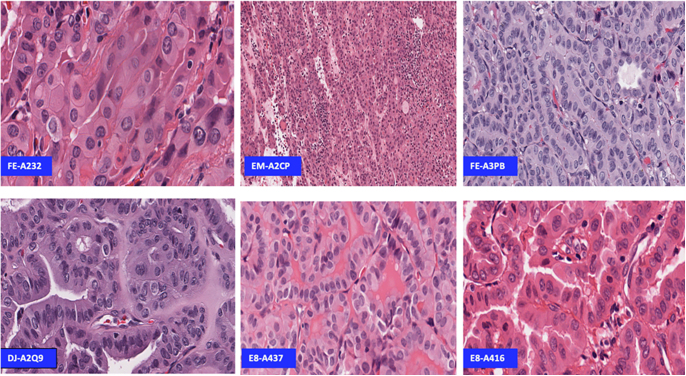
*DICER1* germline-positive cases of the TCGA database with oncocytic morphology, with high-quality available tissue images, from the study of Canberk *et al.* 2021 (9).

Only a limited number of studies have documented detailed pathological characteristics focusing on *DICER1*, predominantly prioritising a genotype-first approach. These investigations, such as those detailed by Mirshahi *et al.* (60), have generally emphasised the genetic aspects of *DICER1* mutations, focusing more on their prevalence, penetrance, and phenotypic implications rather than on their comprehensive pathological features. Although earlier reports have linked DICER1 syndrome with oncocytic morphology (61), the first detailed documentation of *DICER1* mutations associated with specific pathological characteristics of oncocytic morphology was published by Wasserman *et al.* (41). Subsequently, our group analysed a publicly available dataset from The Cancer Genome Atlas (TCGA), identifying oncocytic morphology more frequently in cases with *DICER1* alterations: seven out of eighteen germline alterations and two out of three somatic mutation cases exhibited this feature (9), as depicted in Fig. 3. Our phenotype-first approach enabled a detailed characterisation of these malignancies. In our study, nine cases of PTC with ‘protein-altering germline variants’ of *DICER1* included three oncocytic, two follicular, two classical, and one hobnail (9). While our data identified an appreciable fraction of *DICER1*‐mutated tumours with oncocytic features, we acknowledge that these observations are based on a relatively small sample size and do not achieve formal statistical significance. Accordingly, our findings should be viewed as preliminary and require confirmation in larger studies. In our analysis of the TCGA dataset, we observed that bilaterality was significantly more frequent in *DICER1*-mutated PTCs, including both syndromic and non-syndromic cases. While bilaterality in PTC has been associated with an increased risk of central lymph node metastasis and recurrence (60, 61), we emphasise that this observation applies to the subset of PTCs studied and should not be interpreted as a generalisable feature of all *DICER1*-associated thyroid tumours, such as FTC or IEFVPTC (62, 63).

Notably, all *DICER1* germline variants in our study co-occurred with canonical TC mutations, such as *BRAF V600E* and one *CCDC6-RET* fusion, without loss of the second allele. This pattern supported the mice model of Kumar *et al.* (35), where *DICER1* may function through haploinsufficiency. Such findings highlight the potential of exploring *DICER1* as a future target in novel cancer therapies, though currently no specific therapies targeting *DICER1* mutations are clinically available (3, 64). Indeed, genome- and phenotype-first approaches are not mutually exclusive but complementary to fill the gap from clinical manifestation to therapeutic application and may refine the management of monogenic disorders through establishing reliable consensus testing and surveillance recommendations (65). *DICER1* mutations identified in thyroid neoplasms were grouped under several distinct diagnoses. These diagnoses include PTC with focal hobnail and tall cell changes, oncocytic subtype of PTC, hobnail subtype of PTC, OCA (then referred to as Hürthle cell carcinoma, HCC), tall cell subtype of PTC, and even more aggressive cases of anaplastic thyroid carcinoma (ATC) arising from pre-existing differentiated thyroid carcinoma with oncocytic features, such as PTC tall cell subtype and OCA (Table 2) (9, 23, 41, 50). Moreover, a study from Poiana *et al.* (61) reported the presence of a PTC with oncocytic features accompanied by Sertoli–Leydig cell tumour (SLCT), along with a case reported by Shin *et al.* (66) of an FTC with oncocytic features along with pleuropulmonary blastoma (PPB). The presence of these tumours is highly suggestive of DICER1 syndrome, as a study conducted by Schultz *et al.* (67) in 2024 reported; however, the molecular profile of *DICER1* was not assessed in those studies.

**Table 2:** Thyroid lesions that exhibit oncocytic^†^ features, harbouring different *DICER1* mutations, both somatic and germline.

Thyroid lesions	Age	Other lesions	*DICER1* mutations	Germline vs somatic origin	Reference
PTC with focal hobnail and tall cell changes	10	-	c.4260_4262delGGA (p. E1420del)	Not known	(41)
OV-PTC (TCGA-E8-A416)	51	PPB; CPS	c.4680G>A (p.Ala1560Ala)	Germline	(9)
OV-PTC (TCGA-DE-A0XZ)	65	PPB; CPS	c.4891T>G (p.Ser1631Ala)	Germline	(9)
OV-PTC (TCGA-E8-A437)	27	PPB; CPS	c.2557A>G (p.Ile853Val)	Germline	(9)
OV-PTC (TCGA-DJ-A2Q9)	65	-	c.59C>T (p.Ala20Val)	Germline	(9)
OV-PTC (TCGA-FE-A232)	44	PPB; CPS	c.20A>G (p.Gln7Arg)	Germline	(9)
OV-PTC (TCGA-EM-A2CP)	26	PPB; CPS	c.5013G>C (p.Lys1671Asn)	Germline	(9)
HV-PTC (TCGA-FE-A3PB)	33	PPB; CPS	c.3778G>A (p.Val1260Ile)	Germline	(9)
OV-PTC (TCGA-EM-A2CT)	20	-	c.5718A>C (p.R1906S)	Somatic	(9)
OV-PTC (TCGA-EL-A3D5)	44	-	c.5438A>G (p.E1813G)	Somatic	(9)
OCA*	NA	-	c.20A>G (p. Q7R)	Germline	(23)
OCA*	74	-	(p.E328K)	Somatic	(50)
OCA*	68	-	(p.P750S)	Somatic	(50)
Tall cell variant of PTC	58	-	(p.Q84*)	Somatic	(50)
Tall cell variant of PTC	82	-	(p.L179F)	Somatic	(50)
Tall cell variant of PTC	53	-	(p.H341Y)	Somatic	(50)
PTC tall cell phenotype	67	-	(p.V1740F)	Somatic	(50)
ATC with PTC tall cell variant	81	-	(p.E1800Q)	Somatic	(50)
ATC with OCA*	65	-	(p.M1402T)	Somatic	(50)

* Then referred to as Hürthle cell carcinoma (HCC) in these articles.

^†^ We verified oncocytic morphology in our cases and in the cited hobnail series by direct figure review. ATC is listed solely to document dedifferentiation from oncocytic or tall-cell precursors. PTC, papillary thyroid carcinoma; NA, not available; OV, oncocytic variant; PPB, pleuropulmonary blastoma; CPS, cancer predisposition syndrome; HV, hobnail variant; ATC, anaplastic thyroid carcinoma; HCC, Hürthle cell carcinoma; OCA, oncocytic carcinoma.

Beyond PTC, Pinto *et al.* (68) also reported FTA with oncocytic morphology. The scarcity of cases mentioning oncocytic morphology may be due to a lack of detailed focus on this histotype, as oncocytic lesions are often overlooked. We believe that a re-evaluation of these cases could potentially reveal an increased number of *DICER1*-associated lesions with oncocytic morphology (69).

The miRNA biogenesis pathway is closely linked to epigenetic modifications, particularly methylation patterns, due to miRNAs targeting enzymes that regulate these processes (68). Ten-eleven translocation (TET) enzymes play a key role in this by converting 5-methylcytosine (5mC) to 5-hydroxymethylcytosine (5hmC), which is crucial for DNA demethylation and gene regulation (70). High levels of 5hmC are found in the 5′ untranslated regions (UTRs) of genes involved in miRNA biogenesis, namely *DICER1* and Argonaute RISC Catalytic Component 2 *(AGO2*) (69, 71). Dysregulation of 5hmC, often associated with somatic mutations such as those in *TETs,* DNA methyltransferases *(DNMTs),* and isocitrate dehydrogenases *(IDHs)*, is linked to mitochondrial dysfunction and oncocytic features (70, 72). Our research has shown lower 5hmC levels in thyroid tumours, indicating a hypermethylation pattern, including in oncocytic tumours (14). In addition, Burger *et al.* (73) described a non-canonical *DICER1* isoform that functions within the nucleus and promotes a hypermethylated environment, suggesting that both canonical and non-canonical *DICER1* mechanisms may contribute to hypermethylation in thyroid lesions. In conjunction with miRNA processing, *DICER1* is also involved in DNA processing, chromatin structure remodelling, and apoptosis (25). Recent studies have shown that a reduction in *DICER1* expression levels results in a more open chromatin structure, characterised by decreased methylation, increased histone acetylation, and the loss of chromatin-bound AGO proteins (74, 75). In thyroid malignancies, including those with oncocytic features, overexpression of histone deacetylases (HDACs) has been observed, with elevated HDAC levels being linked to important clinicopathological parameters affecting patient management and prognosis (76, 77). Moreover, epigenetic agents such as HDAC inhibitors have been found to regulate *DICER1* and miRNA expression. These findings suggest that epigenetic modifications, particularly involving HDAC overexpression and DICER1 regulation, may be associated with the development of oncocytic characteristics, although a direct mechanistic link has not been demonstrated (78).

Alterations in gene methylation patterns and specific miRNA profiles have been observed in oncocytic tumours, allowing differentiation between benign and malignant forms (24, 69). Nikiforova *et al.* (24) established a miRNA profile for thyroid tumours, including oncocytic adenomas and OCA. OCA have an upregulation of miRs-187, 221, 339, 183, 222, and 197, in contrast to oncocytic adenomas that portray an upregulation of miRs-31, 339, 183, 221, 224, and 203 (24). However, the genetic basis behind these differences between benign and malignant tumours remains unknown. Further research addressing this gap should be performed, since these miRNA changes might stem from disruptions in miRNA biogenesis components such as *DGCR8* and *DICER1*.

While the specific role of miRNAs in oncocytic thyroid lesions in link to *DICER1* is new, there are already emerging studies reporting a direct connection between *DICER1* mutations and OCA. Ghossein *et al.* (50) described the presence of *DICER1* mutations in 2 out of 50 OCA. A downregulation of *DICER1* was observed in a previous study, both at mRNA and protein levels, being especially pronounced in OCA (49). Paulsson *et al.* (49) reported a case of OCA with loss of one copy of *DICER1;* this case displayed low levels of *DICER1* mRNA expression. Those previous findings suggest that not only mutation in *DICER1* but also dysregulation of its expression, could be important in OCA tumorigenesis, it being believed that downregulation of *DICER1* could be an early event in tumorigenesis (49). This growing body of research is supported by a holistic approach with human pathology findings, reinforcing the existence of a link between *DICER1* and miRNAs in oncocytic thyroid tumours (9). However, further investigation from the scientific community is essential to fully understand and confirm this association. Future studies employing dedicated mitochondrial functional assays will be crucial to assess the metabolic consequences of *DICER1*- and *DGCR8*-related miRNA biogenesis disruption in thyroid tumours. Such analyses could offer mechanistic insight into potential bioenergetic alterations associated with these genetic events.

Regarding *DGCR8*, oncocytic features have also been observed. From the TCGA database, two cases were documented with *DGCR8* mutations. Specifically, the case TCGA-EM-A2CR, while not classified as oncocytic, exhibits some oxyphilic changes within the tumour. In addition, Paulsson *et al.* (12) recently reported the presence of *DGCR8* LOH in an OCA.

Oncocytic thyroid tumours, as summarised by De Luise *et al.* (79), are marked by mitochondrial dysfunction due to mitochondrial DNA (mtDNA) mutations, which partly link to altered hypoxia-inducible factor 1 alpha (HIF-1α) activity and disrupted cellular metabolism (79). Lai *et al.* (80) identified DICER as a key protein interacting with HIF-1α, where under hypoxic conditions, *DICER1* expression is downregulated through HIF-1α-mediated mechanisms. This interaction promotes the ubiquitination and autophagy-mediated degradation of DICER, affecting the maturation of tumour-suppressive miRNAs such as *let*-7 (80). Hypoxia also epigenetically silences the *DICER1* promoter, further reducing its expression and disrupting miRNA biogenesis (81, 82). While these experimental studies establish a compelling link from HIF-1α and hypoxic signalling to reduced DICER1 activity, it should be noted that direct evidence for the reverse, that *DICER1* mutations themselves alter HIF-1α function or mediate mitochondrial dysfunction via this pathway, is currently lacking. In oncocytic thyroid tumours, these alterations in *DICER1* may nonetheless exacerbate mitochondrial dysfunction, potentially contributing, at least indirectly, to the development of the oncocytic phenotype. Further experimental work will be needed to clarify the directionality and mechanistic depth of these associations.

### Algorithm to raise suspicion for *DICER1*-related thyroid lesions (adults and children/adolescents)

**Figure 4:**
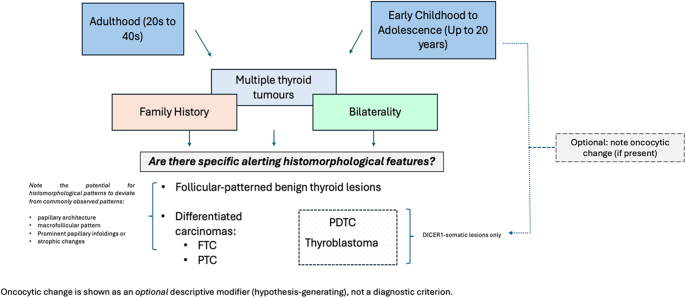
Algorithm to raise suspicion for *DICER1*-related thyroid lesions in adulthood and in early childhood/adolescence. Oncocytic morphology is depicted as an exploratory modifier, not a defining diagnostic feature.

Specific criteria can increase the accuracy of identifying patients with *DICER1* mutation in clinical practice (26). Evaluation of *DICER1* mutations could be beneficial in cases of more aggressive tumours occurring at younger ages, since *DICER1* mutations are associated with early-onset follicular-patterned neoplasms and PDTC (39, 43, 83). Condello *et al.* (26) proposed an algorithm for *DICER1* screening in thyroid lesions based on clinical and histological criteria. Facing a histological diagnosis of FND in younger patients with prominent papillary infoldings, a somatic or constitutional *DICER1* mutation should be screened (83). In the presence of FTA or differentiated thyroid carcinoma with macrofollicular appearance and papillary infoldings with atrophic changes, somatic *DICER1* involvement or somatic/constitutional mutation should be suspected, respectively. For high-grade lesions in younger patients, somatic *DICER1* should be screened (26). This proposed algorithm to predict *DICER1* mutations in follicular-patterned thyroid tumours could identify individuals with syndromic forms of the disease, preventing the consequences of a late diagnosis of malignancy (26). In line with our discussion, Fig. 4 presents an exploratory algorithm for recognising *DICER1*‐related thyroid lesions. While the association between *DICER1* mutations and oncocytic morphology remains preliminary, it nonetheless warrants attention. Larger documentations are needed to determine whether oncocytic changes are indeed more frequent in *DICER1*‐mutated neoplasms and to clarify the underlying molecular mechanisms. We stress that this is not a definitive diagnostic tool, but rather a heuristic to prompt more thorough evaluation, especially in younger patients or in cases with suggestive familial history.

As previously referred*, DICER1*-mutated thyroid lesions are associated with younger patients (from the first decade of life until adulthood), so it is suggested that the presence of thyroid lesions in early life (<40 years), even in the absence of familial history, should be considered for *DICER1* screening (4, 26, 53). The presence of another *DICER1*-associated neoplasm should guide the clinician and pathologist to suspect a germline mutation (4).

Consequently, when DICER1 syndrome or *DICER1* alterations are known in an individual, closer surveillance for thyroid lesions, as well as those in other organs, is essential. For these individuals, it is recommended to have annual clinical examinations from birth to age 20 years, chest X-ray and renal ultrasound from birth to age 6 years every 6 months, and thyroid ultrasound every 3 years from age 8 to age 40 (65).

When considering non-proband carriers of *DICER1* germline variants, TC emerges as one of the most common manifestations (84).

As long as FNA cytology is the main screening tool for the management of thyroid disease, a study conducted by Darbinyan *et al.* (85) dissected the cytomorphologic features of *DICER1*-mutated thyroid lesions. In the latter, cytological preparations showed thyroid lesions of follicular derivation with follicular growth pattern. FNA specimens from four out of seven patients with *DICER1* somatic mutations had similar cytomorphological features, presenting moderately cellular microfollicular-patterned aspirates arranged in small crowded groups with a monomorphic population of follicular cells. Follicular cells showed slight size variation, round nuclei, evenly dispersed finely granular chromatin, and small, inconspicuous nucleoli. The remaining three patients showed indeterminate features. A subsequent histopathological examination of corresponding tissue samples confirmed the neoplastic process of follicular cell origin in five out of seven patients (85). Ultimately, the cytomorphologic features of *DICER1*-mutated thyroid lesions, as elucidated by Darbinyan *et al.* (85), demonstrate a predominant follicular growth pattern with moderately cellular microfollicular arrangements, highlighting the potential utility of FNA cytology in identifying and characterising these distinct neoplasms, which may aid in the early detection and management of *DICER1*-associated thyroid pathologies.

Alongside thyroid surveillance, individuals with a known or suspected germline *DICER1* pathogenic mutation should be enrolled in lung and ovarian surveillance for detection of early PPB and SLCT, respectively, as DICER1 syndrome increases the risk of developing these tumours (67). Moreover, sarcomas of the kidney and central nervous system have been identified as rare *DICER1*-related entities, highlighting the importance of genetic screening and close surveillance in all individuals with known or suspected germline *DICER1* mutation (86, 87).

## Conclusion

This review presents a comprehensive analysis of *DICER1* and *DGCR8* mutations in thyroid pathology, highlighting their significance and dissecting their relation to oncocytic morphology. The understanding of *DICER1*’s role has evolved from syndromic contexts to encompass non-syndromic cases, revealing a higher frequency than initially anticipated. Recent investigations have elucidated detailed pathological characteristics, expanding the known morphological spectrum associated with these mutations. The synthesis of documented cases from the literature and original research findings has been pointing to a notable association between *DICER1* mutations and oncocytic features, an aspect previously underexplored. This association underlines the importance of recognising these distinct histomorphological features in clinical practice. To facilitate the identification and management of *DICER1*-related thyroid lesions, this article proposes a practical algorithm for daily pathology practice, serving as a tool for pathologists to triage potential *DICER1*-related cases and enable more targeted genetic testing and patient care.

The scope of this review extends beyond *DICER1* to include *DGCR8* and the broader miRNA maturation axis, acknowledging their potential involvement in thyroid tumorigenesis. This comprehensive analysis not only consolidates current knowledge but also identifies crucial areas for future research. By synthesising data from both syndromic and non-syndromic cases, detailing pathological characteristics, and incorporating our research findings, we aim to unravel the complex relationship between miRNA biogenesis pathway alterations and thyroid pathology, particularly oncocytic morphology.

## Declaration of interest

The authors declare that there is no conflict of interest that could be perceived as prejudicing the impartiality of the work reported.

## Funding

This study was funded by national funds from FCT—Fundação para a Ciência e Tecnologia, I.P., through a doctoral fellowship to LR (2023.03099.BD), a research contract to JV (2022.00276.CEECIND), and the project “The Porto Comprehensive Cancer Center” with the reference NORTE-01-0145-FEDER-072678—Consórcio PORTO.CCC—Porto.Comprehensive Cancer Center Raquel Seruca. Additional funding was obtained from the project “2022-C05IO101-02—Agenda Illiance (Bosch, project no. 46)—PPS4—OLI health”, with reference C644919832-00000035, funded by PRR—Plano de Recuperação e Resiliência e pelos Fundos Europeus NextGenerationEU, através do sistema de incentivos “Agendas para a Inovação Empresarial”.

## Author contribution statement

LR, RSM, JV, and SC wrote the main manuscript text. All authors provided feedback and supervision. LR created Figs 1 and 2. LR, RSM, JV, and SC created Tables 1 and 2. RSM, SC, and VN created Figs 3 and 4. The Cancer Signalling and Metabolism Research Group of i3s provided the budget for BioRender to create Fig. 1.

## References

[1] Baloch ZW, Asa SL, Barletta JA, et al. Overview of the 2022 WHO classification of thyroid neoplasms. Endocr Pathol 2022 33 27–63. (10.1007/s12022-022-09707-3) 35288841

[2] Nosé V, Gill A, Teijeiro JMC, et al. Overview of the 2022 WHO classification of familial endocrine tumor syndromes. Endocr Pathol 2022 33 197–227. (10.1007/s12022-022-09705-5) 35285003

[3] Canberk S, Correia M, Lima AR, et al. The multifaceted profile of thyroid disease in the background of DICER1 germline and somatic mutations: then, now and future perspectives. J Mol Pathol 2022 3 1–14. (10.3390/jmp3010001)

[4] Riascos MC, Huynh A, Faquin WC, et al. Expanding our knowledge of DICER1 gene alterations and their role in thyroid diseases. Cancers 2024 16 347. (10.3390/cancers16020347) 38254836 PMC10814847

[5] Ali Syeda Z, Langden SSS, Munkhzul C, et al. Regulatory mechanism of microRNA expression in cancer. Int J Mol Sci 2020 21 1723. (10.3390/ijms21051723) 32138313 PMC7084905

[6] Adler D, Lindstrot A, Ochsenfahrt J, et al. Epigenetics-related genes in prostate cancer: expression profile in prostate cancer tissues, androgen-sensitive and -insensitive cell lines. Int J Mol Med 2013 31 21–25. 23135352 10.3892/ijmm.2012.1173 PMC3573772

[7] Hill DA, Ivanovich J, Priest JR, et al. DICER1 mutations in familial pleuropulmonary blastoma. Science 2009 325 965. (10.1126/science.1174334) 19556464 PMC3098036

[8] Rio Frio T, Bahubeshi A, Kanellopoulou C, et al. DICER1 mutations in familial multinodular goiter with and without ovarian Sertoli–Leydig cell tumors. JAMA 2011 305 68–77. (10.1001/jama.2010.1910) 21205968 PMC3406486

[9] Canberk S, Ferreira JC, Pereira L, et al. Analyzing the role of DICER1 germline variations in papillary thyroid carcinoma. Eur Thyroid J 2021 9 296–303. (10.1159/000509183) 33718253 PMC7923931

[10] Oliver‐Petit I, Bertozzi AI, Grunenwald S, et al. Multinodular goitre is a gateway for molecular testing of DICER1 syndrome. Clin Endocrinol 2019 91 669–675. (10.1111/cen.14074) 31408196

[11] Rivera B, Nadaf J, Fahiminiya S, et al. DGCR8 microprocessor defect characterizes familial multinodular goiter with schwannomatosis. J Clin Investig 2020 130 1479–1490. (10.1172/jci130206) 31805011 PMC7269565

[12] Paulsson JO, Rafati N, DiLorenzo S, et al. Whole-genome sequencing of follicular thyroid carcinomas reveal recurrent mutations in MicroRNA processing subunit DGCR8. J Clin Endocrinol Metab 2021 106 3265–3282. (10.1210/clinem/dgab471) 34171097 PMC8530729

[13] Rodrigues L, Canberk S, Macedo S, et al. DGCR8 microprocessor subunit mutation and expression deregulation in thyroid lesions. Int J Mol Sci 2022 23 14812. (10.3390/ijms232314812) 36499151 PMC9740158

[14] Canberk S, Goncalves J, Rios E, et al. The role of 5-hydroxymethylcytosine as a potential epigenetic biomarker in a large series of thyroid neoplasms. Endocr Pathol 2024 35 25–39. (10.1007/s12022-024-09800-9) 38285158 PMC10944390

[15] Krol J, Loedige I & Filipowicz W. The widespread regulation of microRNA biogenesis, function and decay. Nat Rev Genet 2010 11 597–610. (10.1038/nrg2843) 20661255

[16] Chen PS, Lin SC & Tsai SJ. Complexity in regulating microRNA biogenesis in cancer. Exp Biol Med 2020 245 395–401. (10.1177/1535370220907314) PMC7082889 32075432

[17] Winter J & Diederichs S. MicroRNA biogenesis and cancer. Methods Mol Biol 2011 676 3–22. (10.1007/978-1-60761-863-8_1) 20931386

[18] Kusenda B, Mraz M, Mayer J, et al. MicroRNA biogenesis, functionality and cancer relevance. Biomed Pap Med Fac Univ Palacky Olomouc Czech Repub 2006 150 205–215. (10.5507/bp.2006.029) 17426780

[19] Lu J, Getz G, Miska EA, et al. MicroRNA expression profiles classify human cancers. Nature 2005 435 834–838. (10.1038/nature03702) 15944708

[20] Volinia S, Calin GA, Liu CG, et al. A microRNA expression signature of human solid tumors defines cancer gene targets. Proc Natl Acad Sci U S A 2006 103 2257–2261. (10.1073/pnas.0510565103) 16461460 PMC1413718

[21] Paroo Z, Ye X, Chen S, et al. Phosphorylation of the human microRNA-generating complex mediates MAPK/Erk signaling. Cell 2009 139 112–122. (10.1016/j.cell.2009.06.044) 19804757 PMC2760040

[22] Sun HL, Cui R, Zhou J, et al. ERK activation globally downregulates miRNAs through phosphorylating exportin-5. Cancer Cell 2016 30 723–736. (10.1016/j.ccell.2016.10.001) 27846390 PMC5127275

[23] Babazadeh NT, Sinclair TJ, Krishnamurthy V, et al. Thyroid nodule molecular profiling: the clinical utility of Afirma Xpression Atlas for nodules with Afirma Genomic Sequencing Classifier-suspicious results. Surgery 2022 171 155–159. (10.1016/j.surg.2021.08.058) 34924179

[24] Nikiforova MN, Tseng GC, Steward D, et al. MicroRNA expression profiling of thyroid tumors: biological significance and diagnostic utility. J Clin Endocrinol Metab 2008 93 1600–1608. (10.1210/jc.2007-2696) 18270258 PMC2386678

[25] Vergani-Junior CA, Tonon-da-Silva G, Inan MD, et al. DICER: structure, function, and regulation. Biophys Rev 2021 13 1081–1090. (10.1007/s12551-021-00902-w) 35059029 PMC8724510

[26] Condello V, Roberts JW, Stenman A, et al. Atrophic changes in thyroid tumors are strong indicators of underlying DICER1 mutations: a bi-institutional genotype-phenotype correlation study. Virchows Arch 2024 485 105–114. (10.1007/s00428-024-03802-y) 38637342 PMC11271315

[27] Kim J, Field A, Schultz KAP, et al. The prevalence of DICER1 pathogenic variation in population databases. Int J Cancer 2017 141 2030–2036. (10.1002/ijc.30907) 28748527 PMC5749397

[28] de Kock L, Wu MK & Foulkes WD. Ten years of DICER1 mutations: provenance, distribution, and associated phenotypes. Hum Mutat 2019 40 1939–1953. (10.1002/humu.23877) 31342592

[29] Ramírez-Moya J, Wert-Lamas L, Riesco-Eizaguirre G, et al. Impaired microRNA processing by DICER1 downregulation endows thyroid cancer with increased aggressiveness. Oncogene 2019 38 5486–5499. (10.1038/s41388-019-0804-8) 30967628 PMC6755984

[30] Pontén E, Frisk S, Taylan F, et al. A complex DICER1 syndrome phenotype associated with a germline pathogenic variant affecting the RNase IIIa domain of DICER1. J Med Genet 2022 59 141–146. (10.1136/jmedgenet-2020-107385) 33208384 PMC8788248

[31] Lee YY, Lee H, Kim H, et al. Structure of the human DICER-pre-miRNA complex in a dicing state. Nature 2023 615 331–338. (10.1038/s41586-023-05723-3) 36813958

[32] Brenneman M, Field A, Yang J, et al. Temporal order of RNase IIIb and loss-of-function mutations during development determines phenotype in pleuropulmonary blastoma/DICER1 syndrome: a unique variant of the two-hit tumor suppression model. F1000Res 2015 4 214. (10.12688/f1000research.6746.2) 26925222 PMC4712775

[33] Juhlin CC. On the chopping block: overview of DICER1 mutations in endocrine and neuroendocrine neoplasms. Surg Pathol Clin 2023 16 107–118. (10.1016/j.path.2022.09.010) 36739158

[34] Khan NE, Bauer AJ, Schultz KAP, et al. Quantification of thyroid cancer and multinodular goiter risk in the DICER1 syndrome: a family-based cohort study. J Clin Endocrinol Metab 2017 102 1614–1622. (10.1210/jc.2016-2954) 28323992 PMC5443331

[35] Kumar MS, Pester RE, Chen CY, et al. Dicer1 functions as a haploinsufficient tumor suppressor. Genes Dev 2009 23 2700–2704. (10.1101/gad.1848209) 19903759 PMC2788328

[36] Gullo I, Batista R, Rodrigues-Pereira P, et al. Multinodular goiter progression toward malignancy in a case of DICER1 syndrome: histologic and molecular alterations. Am J Clin Pathol 2018 149 379–386. (10.1093/ajcp/aqy004) 29538609

[37] de Kock L, Bah I, Revil T, et al. Deep sequencing reveals spatially distributed distinct hot spot mutations in DICER1-related multinodular goiter. J Clin Endocrinol Metab 2016 101 3637–3645. (10.1210/jc.2016-1328) 27459524

[38] Kim J, Haley J, Hatton JN, et al. A genome-first approach to characterize DICER1 pathogenic variant prevalence, penetrance and cancer, thyroid, and other phenotypes in 2 population-scale cohorts. Genet Med Open 2024 2 101846. (10.1016/j.gimo.2024.101846) 39070603 PMC11271802

[39] Onder S, Mete O, Yilmaz I, et al. DICER1 mutations occur in more than one-third of follicular-patterned pediatric papillary thyroid carcinomas and correlate with a low-risk disease and female gender predilection. Endocr Pathol 2022 33 437–445. (10.1007/s12022-022-09736-y) 36251117

[40] Gallant JN, Chen SC, Ortega CA, et al. Evaluation of the molecular landscape of pediatric thyroid nodules and use of a multigene genomic classifier in children. JAMA Oncol 2022 8 1323–1327. (10.1001/jamaoncol.2022.1655) 35679040 PMC9185516

[41] Wasserman JD, Sabbaghian N, Fahiminiya S, et al. DICER1 mutations are frequent in adolescent-onset papillary thyroid carcinoma. J Clin Endocrinol Metab 2018 103 2009–2015. (10.1210/jc.2017-02698) 29474644

[42] Bae JS, Jung SH, Hirokawa M, et al. High prevalence of DICER1 mutations and low frequency of gene fusions in pediatric follicular-patterned tumors of the thyroid. Endocr Pathol 2021 32 336–346. (10.1007/s12022-021-09688-9) 34313965

[43] Chernock RD, Rivera B, Borrelli N, et al. Poorly differentiated thyroid carcinoma of childhood and adolescence: a distinct entity characterized by DICER1 mutations. Mod Pathol 2020 33 1264–1274. (10.1038/s41379-020-0458-7) 31937902 PMC7329587

[44] Yegen G, Altay AY, Yılmaz İ, et al. DICER1 mutations do not always indicate dismal prognosis in pediatric poorly differentiated thyroid carcinomas. Endocr Pathol 2023 34 279–286. (10.1007/s12022-023-09780-2) 37574466

[45] Ver Berne J, Van den Bruel A, Vermeire S, et al. DICER1 mutations define the landscape of poorly differentiated thyroid carcinoma in children and young adults: case report and literature review. Am J Surg Pathol 2024 48 1277–1283. (10.1097/pas.0000000000002265) 38912716

[46] Agaimy A, Witkowski L, Stoehr R, et al. Malignant teratoid tumor of the thyroid gland: an aggressive primitive multiphenotypic malignancy showing organotypical elements and frequent DICER1 alterations-is the term “thyroblastoma” more appropriate? Virchows Arch 2020 477 787–798. (10.1007/s00428-020-02853-1) 32507920 PMC7683491

[47] Rooper LM, Bynum JP, Miller KP, et al. Recurrent DICER1 hotspot mutations in malignant thyroid gland teratomas: molecular characterization and proposal for a separate classification. Am J Surg Pathol 2020 44 826–833. (10.1097/pas.0000000000001430) 31917706

[48] Rooper LM. From malignant thyroid teratoma to thyroblastoma: evolution of a newly-recognized DICER1 -associated malignancy. Adv Anat Pathol 2023 30 136–145. (10.1097/pap.0000000000000364) 36069850

[49] Paulsson JO, Wang N, Gao J, et al. GABPA-dependent down-regulation of DICER1 in follicular thyroid tumours. Endocr Relat Cancer 2020 27 295–308. (10.1530/erc-19-0446) 32163919 PMC7159166

[50] Ghossein CA, Dogan S, Farhat N, et al. Expanding the spectrum of thyroid carcinoma with somatic DICER1 mutation: a survey of 829 thyroid carcinomas using MSK-IMPACT next-generation sequencing platform. Virchows Arch 2022 480 293–302. (10.1007/s00428-021-03212-4) 34580763 PMC10126990

[51] Schultz KAP, MacFarland SP, Perrino MR, et al. Update on pediatric surveillance recommendations for PTEN hamartoma tumor syndrome, DICER1-related tumor predisposition, and tuberous sclerosis complex. Clin Cancer Res 2025 31 234–244. (10.1158/1078-0432.ccr-24-1947) 39540884 PMC11747828

[52] Lee YA, Im SW, Jung KC, et al. Predominant DICER1 pathogenic variants in pediatric follicular thyroid carcinomas. Thyroid 2020 30 1120–1131. (10.1089/thy.2019.0233) 32228164

[53] Mastnikova K, Bulanova Pekova B, Kuklikova V, et al. DICER1 variants in pediatric and young adult thyroid nodules. Thyroid 2024 34 1225–1233. (10.1089/thy.2024.0188) 39283830

[54] Rutter MM, Jha P, Schultz KA, et al. DICER1 mutations and differentiated thyroid carcinoma: evidence of a direct association. J Clin Endocrinol Metab 2016 101 1–5. (10.1210/jc.2015-2169) 26555935 PMC4701837

[55] Han Y, Li S, Zhao B, et al. Clinical, molecular and radiological characteristics of thyroid nodules with somatic DICER1 mutations in adults. Endocr Connect 2025 14 e250125. (10.1530/ec-25-0125) 40530998 PMC12217457

[56] Ricarte-Filho JC, Casado-Medrano V, Reichenberger E, et al. DICER1 RNase IIIb domain mutations trigger widespread miRNA dysregulation and MAPK activation in pediatric thyroid cancer. Front Endocrinol 2023 14 1083382. (10.3389/fendo.2023.1083382) PMC9990750 36896180

[57] Perdas E, Stawski R, Nowak D, et al. The role of miRNA in papillary thyroid cancer in the context of miRNA Let-7 family. Int J Mol Sci 2016 17 909. (10.3390/ijms17060909) 27314338 PMC4926443

[58] Rodrigues L, Da Cruz Paula A, Soares P, et al. Unraveling the significance of DGCR8 and miRNAs in thyroid carcinoma. Cells 2024 13 561. (10.3390/cells13070561) 38607000 PMC11011343

[59] Kim J, Park WJ, Jeong KJ, et al. Racial differences in expression levels of miRNA machinery-related genes, dicer, drosha, DGCR8, and AGO2, in Asian Korean papillary thyroid carcinoma and comparative validation using the cancer genome atlas. Int J Genomics 2017 2017 5789769. (10.1155/2017/5789769) 28352639 PMC5352891

[60] Mirshahi UL, Kim J, Best AF, et al. A genome-first approach to characterize DICER1 pathogenic variant prevalence, penetrance, and phenotype. JAMA Netw Open 2021 4 e210112. (10.1001/jamanetworkopen.2021.0112) 33630087 PMC7907958

[61] Poiana C, Virtej I, Carsote M, et al. Virilising Sertoli–Leydig cell tumour associated with thyroid papillary carcinoma: case report and general considerations. Gynecol Endocrinol 2010 26 617–622. (10.3109/09513591003686361) 20632913

[62] Wang W, Su X, He K, et al. Comparison of the clinicopathologic features and prognosis of bilateral versus unilateral multifocal papillary thyroid cancer: an updated study with more than 2000 consecutive patients. Cancer 2016 122 198–206. (10.1002/cncr.29689) 26506214

[63] Sancho JJ, Lennard TW, Paunovic I, et al. Prophylactic central neck disection in papillary thyroid cancer: a consensus report of the European Society of Endocrine Surgeons (ESES). Langenbecks Arch Surg 2014 399 155–163. (10.1007/s00423-013-1152-8) 24352594

[64] Cazzato G, Casatta N, Lupo C, et al. DICER1 tumor syndrome: a retrospective review and future perspectives. J Mol Pathol 2024 5 264–275. (10.3390/jmp5030019)

[65] Bakhuizen JJ, Hanson H, van der Tuin K, et al. Surveillance recommendations for DICER1 pathogenic variant carriers: a report from the SIOPE Host Genome Working Group and CanGene-CanVar Clinical Guideline Working Group. Fam Cancer 2021 20 337–348. (10.1007/s10689-021-00264-y) 34170462 PMC8484187

[66] Shin SH, Yoon JH, Son MH, et al. Follicular thyroid carcinoma arising after hematopoietic stem cell transplantation in a child with pleuropulmonary blastoma. Thyroid 2012 22 547–551. (10.1089/thy.2011.0161) 22468940

[67] Schultz KAP, Nelson AT, Mallinger PHR, et al. DICER1-Related tumor predisposition: identification of at-risk individuals and recommended surveillance strategies. Clin Cancer Res 2024 30 5681–5692. (10.1158/1078-0432.ccr-24-1532) 39400264 PMC11649450

[68] Pinto AE, Silva GL, Henrique R, et al. Familial vs sporadic papillary thyroid carcinoma: a matched-case comparative study showing similar clinical/prognostic behaviour. Eur J Endocrinol 2014 170 321–327. (10.1530/eje-13-0865) 24272198

[69] Canberk S, Lima AR, Correia M, et al. Oncocytic thyroid neoplasms: from histology to molecular biology. Diagn Histopathol 2019 25 154–165. (10.1016/j.mpdhp.2019.02.002)

[70] Tan L & Shi YG. Tet family proteins and 5-hydroxymethylcytosine in development and disease. Development 2012 139 1895–1902. (10.1242/dev.070771) 22569552 PMC3347683

[71] Azizgolshani N, Petersen CL, Chen Y, et al. DNA 5-hydroxymethylcytosine in pediatric central nervous system tumors may impact tumor classification and is a positive prognostic marker. Clin Epigenetics 2021 13 176. (10.1186/s13148-021-01156-9) 34538273 PMC8451154

[72] Oishi N, Vuong HG, Mochizuki K, et al. Loss of 5-Hydroxymethylcytosine is an epigenetic hallmark of thyroid carcinomas with TERT promoter mutations. Endocr Pathol 2020 31 359–366. (10.1007/s12022-020-09652-z) 33058026

[73] Burger K & Gullerova M. Swiss army knives: non-canonical functions of nuclear Drosha and Dicer. Nat Rev Mol Cell Biol 2015 16 417–430. (10.1038/nrm3994) 26016561

[74] Ameyar-Zazoua M, Rachez C, Souidi M, et al. Argonaute proteins couple chromatin silencing to alternative splicing. Nat Struct Mol Biol 2012 19 998–1004. (10.1038/nsmb.2373) 22961379

[75] Haussecker D & Proudfoot NJ. Dicer-dependent turnover of intergenic transcripts from the human beta-globin gene cluster. Mol Cell Biol 2005 25 9724–9733. (10.1128/mcb.25.21.9724-9733.2005) 16227618 PMC1265824

[76] Canberk S, Lima AR, Pinto M, et al. Epigenomics in hurthle cell neoplasms: filling in the gaps towards clinical application. Front Endocrinol 2021 12 674666. (10.3389/fendo.2021.674666) PMC8181423 34108939

[77] Jung CK, Kim Y, Jeon S, et al. Clinical utility of EZH1 mutations in the diagnosis of follicular-patterned thyroid tumors. Hum Pathol 2018 81 9–17. (10.1016/j.humpath.2018.04.018) 29723601

[78] Hoffend NC, Magner WJ & Tomasi TB. The epigenetic regulation of dicer and microRNA biogenesis by panobinostat. Epigenetics 2017 12 105–112. (10.1080/15592294.2016.1267886) 27935420 PMC5330440

[79] De Luise M, Girolimetti G, Okere B, et al. Molecular and metabolic features of oncocytomas: seeking the blueprints of indolent cancers. Biochim Biophys Acta Bioenerg 2017 1858 591–601. (10.1016/j.bbabio.2017.01.009) 28115060

[80] Lai HH, Li JN, Wang MY, et al. HIF-1α promotes autophagic proteolysis of dicer and enhances tumor metastasis. J Clin Investig 2018 128 625–643. (10.1172/jci89212) 29251629 PMC5785260

[81] Ting AH, Suzuki H, Cope L, et al. A requirement for DICER to maintain full promoter CpG island hypermethylation in human cancer cells. Cancer Res 2008 68 2570–2575. (10.1158/0008-5472.can-07-6405) 18413723 PMC2828041

[82] van den Beucken T, Koch E, Chu K, et al. Hypoxia promotes stem cell phenotypes and poor prognosis through epigenetic regulation of DICER. Nat Commun 2014 5 5203. (10.1038/ncomms6203) 25351418 PMC4255228

[83] Jung CK, Liu Z, Hirokawa M, et al. Histological clues of DICER1 mutations in thyroid nodules. Virchows Arch 2024 485 755–757. (10.1007/s00428-024-03915-4) 39231821

[84] Stewart DR, Best AF, Williams GM, et al. Neoplasm risk among individuals with a pathogenic germline variant in DICER1. J Clin Oncol 2019 37 668–676. (10.1200/jco.2018.78.4678) 30715996 PMC6553836

[85] Darbinyan A, Morotti R, Cai G, et al. Cytomorphologic features of thyroid disease in patients with DICER1 mutations: a report of cytology-histopathology correlation in 7 patients. Cancer Cytopathol 2020 128 746–756. (10.1002/cncy.22329) 32897650

[86] Schoettler PJ, Smith CC, Nishitani M, et al. Anaplastic sarcoma of the kidney (DICER1-sarcoma of the kidney): a report from the International Pleuropulmonary Blastoma/DICER1 registry. Pediatr Blood Cancer 2024 71 e31090. (10.1002/pbc.31090) 38807260 PMC11590164

[87] Eldaya RW, Fagan RJ, Dagher SA, et al. Imaging features of primary intracranial sarcoma with DICER1 mutation: a multicenter case series. AJNR Am J Neuroradiol 2024 45 626–631. (10.3174/ajnr.a8192) 38637027 PMC11288540

